# Milk Diet for Gout

**Published:** 1886-03

**Authors:** 


### MILK DIET FOR GOUT.

There has been merriment in the old halls of England about honest Hodge and the cow. I am almost a Hindu in my veneration for that animal, whom I regard as the foster-mother of mankind. The doctors are beginning to discover her value, not only for babes, but for adults. But house-fed cows are only good for the undertaker's trade, because they are sure to diffuse consumption. The other day I went to see an English friend who had been suffering from a sharp attack of gout. He was taken with it here, on his way to Cannes, and thought himself too ill to recover. There were twinges everywhere. Each essential organ becomes in turn the seat of the disease, and his spirits were depressed and his brain foggy. An ordinary doctor mildly suggested a lacteal diet, but as the patient hated milk, he refused to accept this regimen. A specialist was then called in. He said, "Meat and wine—not excepting Bordeaux—are, in your present state, rank poison. You must take no tea, coffee, or any other stimulant for ten days. Your food is to be milk every three hours. If you find it too heavy mix it with Hauterive-Vichy. Vary it, too, by having it mixed with onion or leek soups, or with eggs done *a la creme*. I have had patients who were half suffocated with gouty matter in their blood, and others who thought their brains were softening. This treatment cured them in a short time. Milk is food in its most perfect form, and should be regarded as a staple aliment by old and young." M. Barthelemy St. Hilaire, who is as old as M. Grevy, has lived for years chiefly on milk. He keeps a hornless goat which gives him nearly three quarts a day. An old woman is hired to take the animal out regularly for a walk, and to bring it fresh grass. M. St. Hilaire does not wear a great coat, however inclement the weather. He walks from Passy to the Senate and Institute, when he has business there, and back; has never ridden in a wheeled vehicle since he was Foreign Minister, is free from every infirmity of old age, and gets up every morning at 5 o'clock to work at his translation of "Aristotle," of which he has yet seven volumes to get through. He thinks that most of the ills to which the rich are subject come of eating and drinking too much, and trying to have more than their share of enjoyment.--*Paris Letter to London Truth.*

**The** suggestion is made that bottles containing citrate of silver be stowed away in ships' boats. Seven ounces of the citrate will, it is said, turn enough sea water into drinking water to supply a man for a week.

